# An Innovative Dual-Wavelength Laser Technique for Atrophic Acne Scar Management: A Pilot Study

**DOI:** 10.3390/medicina59112012

**Published:** 2023-11-15

**Authors:** Stefania Belletti, Francesca Madeddu, Giuseppe Fabrizio Amoruso, Eugenio Provenzano, Steven Paul Nisticò, Irene Fusco, Luigi Bennardo

**Affiliations:** 1 Private Practice, Studio Kalos, 20100 Milan, Italy; sbellez@libero.it; 2 El. En. Group, 50041 Calenzano, Italy; f.madeddu@elen.it; 3 Azienda Sanitaria Locale di Crotone, 88816 Strongoli, Italy; amorusogf@gmail.com; 4 Department of Dermatology, Cosenza Hospital, 87100 Cosenza, Italy; e.provenzano@aocs.it; 5 Department of Dermatology, University of Rome “La Sapienza”, 00185 Roma, Italy; steven.nistico@gmail.com; 6 Department of Health Sciences, Magna Graecia University, 88100 Catanzaro, Italy; l.bennardo@unicz.it

**Keywords:** laser, acne scars, atrophic scars

## Abstract

*Background and Objectives*: Acne scars are one of the most disturbing and long-term symptoms of acne vulgaris, having a negative impact on a person’s physical, emotional, and social well-being. *Aim*: the purpose of the study was to evaluate the efficacy and post-treatment outcomes of a dual-wavelength system combining the irradiation of two wavelengths at 10,600 nm and 1540 nm in the management of facial atrophic acne scars. *Materials and Methods*: Four healthy adult volunteers aged 24–53 years were enrolled. The areas treated were the full face (two patients), cheeks (one patient), and forehead (one patient). A dual-wavelength system (1540 nm and 10,600 nm) was used for this study. Patients underwent 2–4 treatment sessions, and the treatments were performed once every 45–90 days. All possible side effects such as burning sensation, dyschromia, mild to moderate post-treatment erythema, bleeding, itching, edema, and crusting were checked. The index to assess edema and erythema was based on a four-point scale (none, mild, moderate, and severe) and was applied before and at 3-month follow-up (3 MFU) after the last treatment session. In addition, a patient assessment was conducted before treatment and at 3 MFU after the last treatment session. *Results*: For all patients examined, the edema index was mild, while for the erythema index, 3/4 patients experienced moderate and 1/4 patients experienced mild symptoms. The mean patient downtime was 5.8 ± 0.5 days. Concerning the patient assessment, 2/4 subjects showed excellent improvement, 1/4 patients showed good improvement, and 1/4 patients showed slight improvement. As shown by the photographic assessment, a noticeable improvement in skin texture and a substantial reduction in acne scars were observed at the end of treatment. *Conclusions*: This dual-wavelength laser technology has the potential to be an interesting and safe approach for acne scar treatment, with a low risk of scarring/hypopigmentation and a shorter healing time.

## 1. Introduction

Acne scars are one of the most disturbing and long-term symptoms of acne vulgaris, having a negative impact on a person’s physical, emotional, and social well-being. Acne scars are classified as atrophic or proliferative [1]. Of these, atrophic scars are more difficult to clinically treat than other types of traumatic scars.

Depending on the clinical condition, there are several options for therapy available, including surgical and non-invasive techniques [2].

The primary forms of treatment still include topical retinoids, benzoyl peroxide, azelaic acid, antibiotics, isotretinoin [3], and chemical peeling (with glycolic acid or lactate acid), but they often result in poor compliance, a lack of long-term remission, and side effects. The application of lasers is continuously rising due to their minimal difficulties with treating acne, the limited number of office-based treatments required, the potential benefits of simultaneously treating acne scarring, and the rapid onset of outcomes.

A 1450 nm diode laser, 585 and 595 nm pulsed dye lasers (PDLs), a 532 nm potassium titanyl phosphate laser, near-infrared diode lasers, a 1320 nm Nd:YAG laser, a 1540 nm erbium (Er):glass laser, a 1550 nm Er:glass fractional laser, a 1064 nm long-pulsed Nd:YAG laser, and ablative laser therapy (using CO_2_ and Er:YAG lasers) represent the most often utilized lasers for treating acne and acne scars [4,5,6,7,8].

Progress in this technology has made lasers available with so-called fractional technology. Thousands of microablative zones (MAZs) and microthermal zones (MTZs) of damage are produced by this type of laser system, and they are surrounded by areas of normal tissue. This allows for concentrating the ablation and thermal stimulation of the laser on the tissue only in correspondence with specific spots separated by areas of undamaged skin. By doing so, it is possible to rejuvenate the skin with a considerable depth of action and with an important thermal stimulation of the connective tissue. Meanwhile, it also reduces aggressiveness compared to traditional ablative laser treatments, therefore reducing or eliminating recovery times. Depending on the type of laser source used, fractional technologies can have an ablative or non-ablative effect: the ablative effect with a fractional CO_2_ laser involves the removal of superficial layers and, therefore, a visible exfoliation and a few days of convalescence, while the non-ablative effect achieved with a 1540 nm fractional laser does not lead to the removal of superficial layers and therefore does not involve convalescence. Several published studies have already demonstrated that the sequential action of CO_2_ and infrared wavelengths—specifically the synergistic sequential emission of fractional 10,600 and 1540 nm wavelengths—extends and enhances the thermal effect. This guarantees longer healing durations for the fractionated emission modes, which increases cell turnover and stimulates deeper into the tissue, resulting in more effective therapies for tissue remodeling [9,10,11,12,13,14,15,16].

By controlling the power and pulse duration (dwell time) parameters, this dual-wavelength (10,600 nm + 1540 nm) system emits perfectly controlled energy/DOT, where DOT represents the columns of thermal damage. Lastly, the “DOT spacing” can be chosen, which determines the area of the tissue not exposed to CO_2_ irradiation [9].

**Figure 1:**
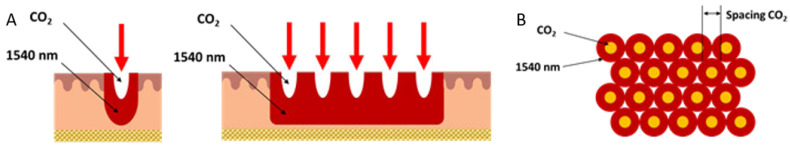
A schematic image representing the two wavelengths’ action to more clearly illustrate the thermal impact and ablation of each laser wavelength, either separately or in combination. Front view (**A**) and top view (**B**). Courtesy of DEKA M.E.LA srl.

Additionally, this method permits several CO_2_ pulse shapes, which are essential for guaranteeing the release of heat deeper into the dermis as well as the superficial ablation of the epidermis. The optical width of the CO_2_ spot is approximately 250 µm. With spots of the order of 1000 µm emitted on the same axis as the DOT and typical CO_2_ spacing parameters (approximately 500 µm) as used in the literature for dermatological ablations, the entire scanning area can be heated uniformly, continuously, and without coagulation thanks to the second wavelength at 1540 nm delivered by the new miniaturized scanning systems. Combining CO_2_ with infrared wavelengths in a sequential manner ensures that the fractionated emission modes heal over time, extending and intensifying the thermal effect for a more successful tissue remodeling process (Figure 1).

This laser technology can promote neocollagenesis and the wound healing process. Fibrin network clots and granulation tissue, which is created by newly deposited collagen, are the primary structures involved in wound healing. The fibrinolytic system typically dissolves clots once they have formed in vivo and completed their hemostatic role in order to restore the compromised blood flow. Indeed, fibrin plug production is a perfectly natural phenomenon that typically occurs before a wound’s complete repair [17]. Recent studies such as that of Kositratna et al. (2015) [18] demonstrated rapid fibrin plug formation, initiating at the bottom of the MTZ lesions, after CO_2_ fractional laser procedures.

Based on these considerations, the current study was performed to assess the effectiveness and results of treatment of facial atrophic acne scars using a dual-wavelength system that combines irradiation at two wavelengths, 10,600 nm and 1540 nm.

## 2. Materials and Methods

Four healthy adult volunteers aged 24–53 years (mean: 38 ± 13), comprising two men and two women affected by atrophic acne scars with a Fitzpatrick skin type ranging between I (3 patients) and II (1 patient), were recruited from December 2022 to March 2023 for this study. The areas treated were the full face (2 patients), cheeks (1 patient), and forehead (1 patient).

Patients with facial CO_2_ fractional resurfacing justifications for medical or aesthetic purposes who desired measurable follow-up after laser therapy were considered eligible for inclusion. The exclusion criteria were as follows: subjects having recently undergone an exfoliation treatment; individuals who had undergone surgical procedures, such as lifting; subjects with a history of herpes or evidence of active herpes simplex virus infection; sun and UV lamp exposure (to be avoided for at least 1 month before) during and after treatment; patients who were taking or had taken retinoids in the 45 days prior to treatment; patients who were taking anticoagulants during the treatment period; photo-sensitizers; pregnancy.

The patients had not previously undergone any other kind of treatment.

A dual-wavelength system (DuoGlide, DEKA M.E.L.A Srl, Florence, Italy) was employed in this research. The system comprises a multi-technology that includes a 10,600 nm CO_2_ laser device and a 1540 nm laser. The scanner’s capacity to provide one or both wavelengths (1540 nm and 10,600 nm) in a sequential emission mode on the same DOT (the area that interests both MAZs and MTZs in the tissue) allows for a customizable balance between ablation and coagulation depths. This enables the delivery of new and more effective treatments. Utilizing the 1540 + CO_2_ sequence, one pass across the specific treated area was performed; thus, both wavelengths simultaneously targeted the skin. For this purpose, treatments were carried out using a fractioned scanning unit (μScan DOT). The laser parameters for the CO_2_ wavelength source were as follows: power, 10–15 watts; dwell time, 900–1000 µs; SP pulse; spacing, 500–700 µm; Stack 1–2; energy/DOT, 20.1–50.7 mJ (for the 1540 nm source, energy/DOT was 15–20 mJ).

Patients underwent 2–4 treatment sessions depending on the severity of the lesions, and the treatments were performed once every 45–90 days. The procedure complied with the Helsinki Declaration’s (1975) ethical standards [19]. The patients provided informed consent after being given information about the involved risk, benefits, and potential therapeutic outcomes. All potential adverse events such as burning sensation, dyschromia, edema, bleeding, mild to moderate post-treatment erythema, itching, and crusting were evaluated.

An edema and erythema index assessment, based on a 4-point scale (1 = none; 2 = mild; 3 = moderate; 4 = severe), was performed before laser treatment and at 3-month follow-up (3 MFU) after the last laser treatment session. In addition, a patient assessment was administered to all subjects before the laser treatment and at 3 MFU after the last laser treatment session, utilizing the following scores: no improvement, slight improvement, moderate improvement, good improvement, and excellent improvement. The days required for the expulsion of the fibrin plugs and the patients’ downtime were monitored in order to evaluate skin recovery. Finally, a photographic assessment was conducted to evaluate the patients’ aesthetic improvement. Black and white photos were chosen to better highlight the texture of the scars before and after laser treatment.

## 3. Results

For all patients examined, the edema index was mild, while for the erythema index, 3/4 patients experienced moderate and 1/4 patient experienced mild symptoms.

The mean patient downtime was 5.8 ± 0.5 days. Concerning the patient assessment, 2/4 subjects showed excellent improvement, 1/4 patients showed good improvement, and 1/4 patients showed slight improvement.

**Figure 2:**
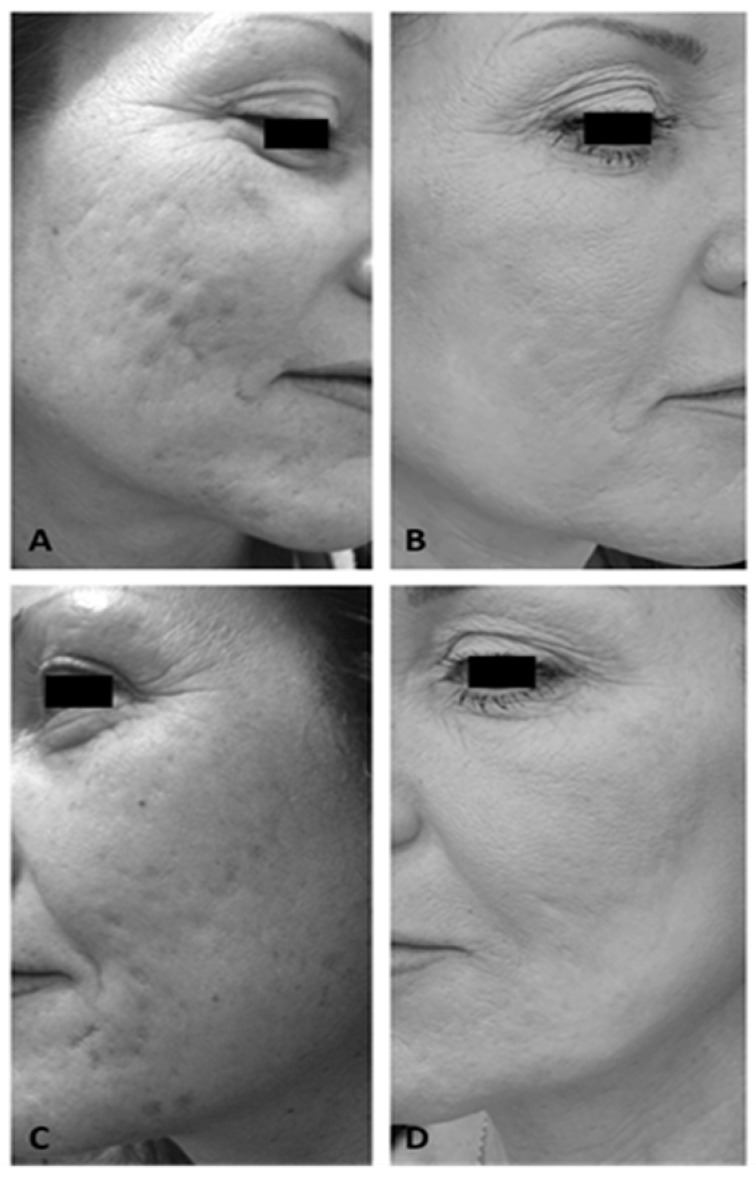
Right lateral view of a female patient’s cheek before (**A**) and after 4 laser treatments (3 MFU after the last treatment session) (**B**). Left lateral view of a female patient’s cheek before (**C**) and after 4 laser treatments (3 MFU after the last treatment session) (**D**).

At the end of the therapy, a significant decrease in atrophic acne scarring and a noticeable improvement in skin texture were noted based on photographic evaluation (3 MFU after the last treatment session) (see Figure 2).

Finally, it was found that after the laser treatment, it took approximately 5 days for the fibrin plugs to be expelled, showing a significant and accelerated scar healing process. No side effects were observed except for slight burning, erythema, and edema, which disappeared within the first 24 h after treatment.

The summary of the results for each patient examined with appropriate descriptions is reported in Table 1.

## 4. Discussion

Our preliminary clinical results, documented by quantitative and qualitative assessments, show a marked reduction in atrophic acne scarring in all patients treated. Thanks to the new energy delivery modality that ensures the superficial layer of the skin is preserved from damage, a rapid healing time was achieved, with an immediate return to normal skin color and sensitivity. Furthermore, the reduced thermal damage ensured no post-treatment residual redness. Indeed, the combined effects of the two wavelengths amplified all the advantages already associated with CO_2_ laser systems. This is accurate in terms of collagen remodeling and stimulation due to a larger volumetric thermal effect, as well as tone strengthening due to a greater shrinkage effect [9]. Indeed, the handpiece used, the µScan DOT, can deliver one or both wavelengths (1540 nm and 10,600 nm) sequentially on the same DOT, allowing for a balance between ablation and coagulation depths as well as the delivery of new and more efficient treatments. Moreover, the scanning area can be heated homogeneously, continuously, and without coagulation using the second wavelength of 1540 nm. Recently published studies demonstrated the potential application of this dual-wavelength laser technique (10,600 nm/1540 nm) for post-surgical scar management, extensive hypertrophic burn scar management, and as an efficient laser-assisted blepharoplasty technique with good and promising results [20,21]. In addition, other recent studies showed that this dual-wavelength laser approach has demonstrated promise as a new and safe nonsurgical solution for skin laxity and rejuvenation [22,23]. With this novel technique, when properly executed, it is possible to obtain results comparable to those with classic CO_2_ laser resurfacing, with an exceptionally minimal risk of scarring and hypopigmentation and a fast healing process.

In our research, the same number of laser sessions (four laser treatments) and the same follow-up (3 months) were selected in comparison to previous published studies, such as that of Mohamed et al. (2018) [24], where both fractional ablative CO_2_ and fractional non-ablative lasers showed good results for acne scar management, with a high efficacy, good safety profile, and good patient satisfaction.

These promising results in the treatment of atrophic acne scars with this new dual-wavelength system need to be greatly corroborated by further investigations with a larger sample of patients and a longer follow-up.

### Study Limitation

The study limitations comprise the small sample of patients and the lack of a longer follow-up. Furthermore, considering that only four patients were examined for this study, another limitation is the lack of a photographic evaluation based on the minimum number of sessions required to improve acne (assessment before four laser treatment sessions).

## 5. Conclusions

Our preliminary clinical data demonstrate that this dual-wavelength laser technique achieved good results in the management of atrophic acne scars in all treated patients, resulting in faster scar healing after treatment. More importantly, this new technology improves the effectiveness of treatment while reducing patient downtime and associated recovery times.

## Figures and Tables

**Table 1:** The summary of the results for each patient examined.

Patients	Sex	Age	Fitzpatrick Skin Type	Treated Area	Number of Sessions	Edema Index	Erythema Index	Expelling of Fibrin Plugs (Days)	Patient’s Assessment
1	Female	53	II	Full face	4	Mild	Moderate	4	Excellent improvement
2	Male	31	I	Cheek	4	Mild	Moderate	4	Excellent improvement
3	Male	42	I	Forehead	3	Mild	Moderate	4	Good improvement
4	Female	24	I	Full face	2	Mild	Mild	4	Slight improvement

## Data Availability

Data that support the study findings are available on request from the corresponding author (I.F.).
